# Synthetic OCT data in challenging conditions: three-dimensional OCT and presence of abnormalities

**DOI:** 10.1007/s11517-021-02469-w

**Published:** 2021-11-18

**Authors:** Hajar Danesh, Keivan Maghooli, Alireza Dehghani, Rahele Kafieh

**Affiliations:** 1 Department of Biomedical Engineering, Science and Research Branch, Islamic Azad University, Tehran, Iran; 2 Isfahan Eye Research Center, Department of Ophthalmology, Isfahan University Of Medical Sciences, Isfahan, Iran; 3 School of Advanced Technologies in Medicine, Medical Image and Signal Processing Research Center, Isfahan University of Medical Sciences, Isfahan, Iran; 4 Biosciences Institute, Newcastle University, Newcastle upon Tyne, UK

**Keywords:** Data augmentation, Optical coherence tomography, Synthetic data

## Abstract

Nowadays, retinal optical coherence tomography (OCT) plays an important role in ophthalmology and automatic analysis of the OCT is of real importance: image denoising facilitates a better diagnosis and image segmentation and classification are undeniably critical in treatment evaluation. Synthetic OCT was recently considered to provide a benchmark for quantitative comparison of automatic algorithms and to be utilized in the training stage of novel solutions based on deep learning. Due to complicated data structure in retinal OCTs, a limited number of delineated OCT datasets are already available in presence of abnormalities; furthermore, the intrinsic three-dimensional (3D) structure of OCT is ignored in many public 2D datasets. We propose a new synthetic method, applicable to 3D data and feasible in presence of abnormalities like diabetic macular edema (DME). In this method, a limited number of OCT data is used during the training step and the Active Shape Model is used to produce synthetic OCTs plus delineation of retinal boundaries and location of abnormalities. Statistical comparison of thickness maps showed that synthetic dataset can be used as a statistically acceptable representative of the original dataset (*p* > 0.05). Visual inspection of the synthesized vessels was also promising. Regarding the texture features of the synthesized datasets, Q-Q plots were used, and even in cases that the points have slightly digressed from the straight line, the *p*-values of the Kolmogorov–Smirnov test rejected the null hypothesis and showed the same distribution in texture features of the real and the synthetic data. The proposed algorithm provides a unique benchmark for comparison of OCT enhancement methods and a tailored augmentation method to overcome the limited number of OCTs in deep learning algorithms.

**Graphical abstract:**

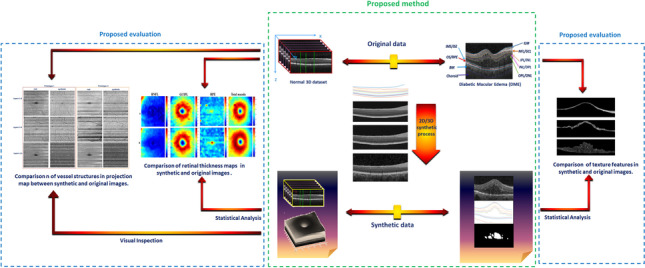

## Introduction

**Fig. 1:**
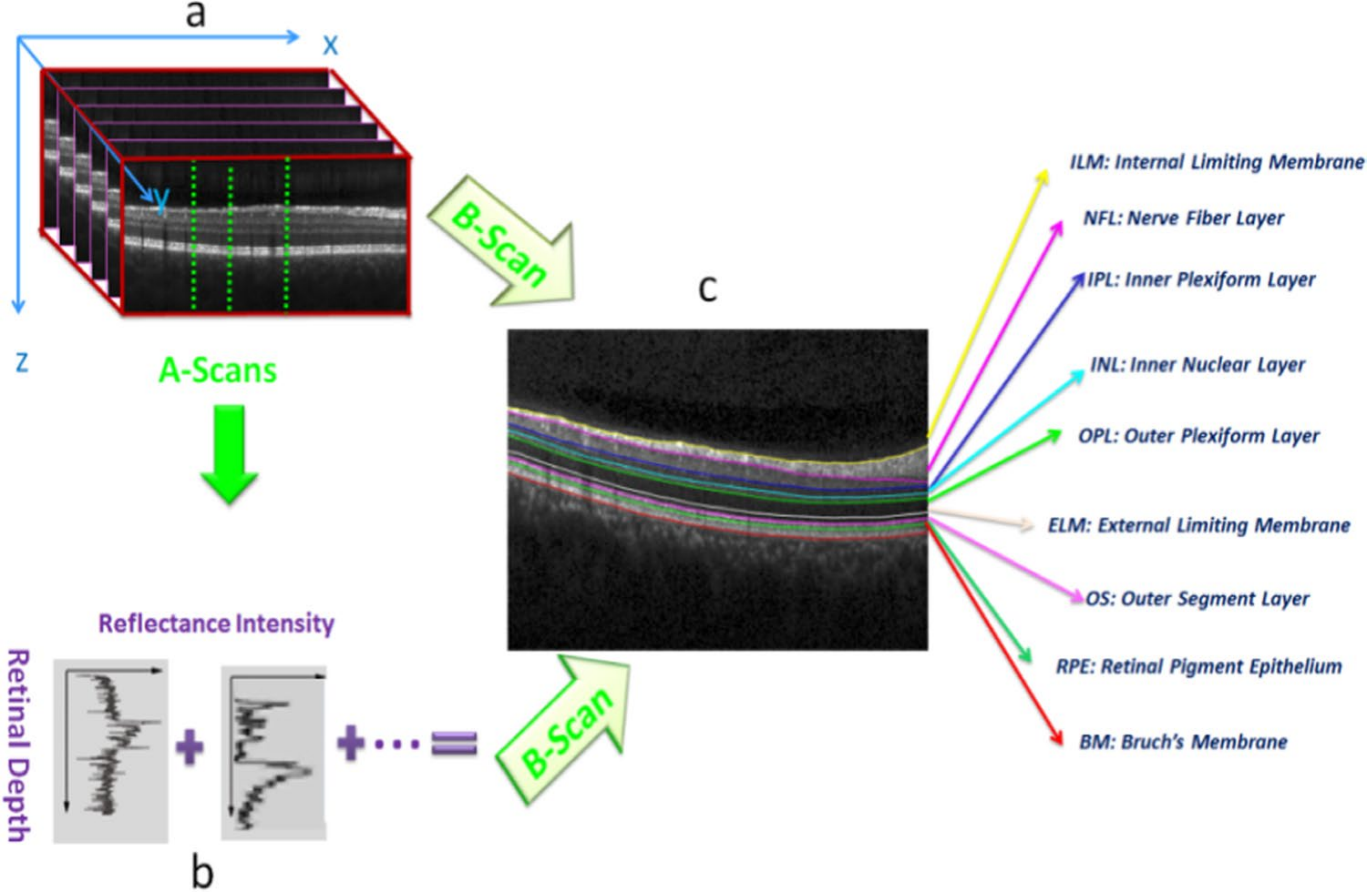
An example of a retinal 3D OCT cube. a) 3D structure with A-scans and B-scans. b) Sample A-scans. c) Retinal layers segmentation on a B-scan

The retina is an important component in the eye, made up of several inter-retinal layers. The primary task of the retina is to convert luminous energy into analyzable signals for the brain [1]. Optical coherence tomography (OCT) is one of the advanced and recent imaging techniques that provide valuable information for ophthalmologists with high-resolution images from transverse cuts of the retina [2]. Stacks of cross-sectional images yield three-dimensional (3D) volumes with detailed data for diagnosis of retinal diseases such as age-related macular degeneration, diabetic retinopathy, glaucoma, diabetic macular edema, etc.[3]. Each 3D OCT volume (Fig. 1a) is composed of cross-sectional images called B-scan (x–z images in Fig. 1c) and each B-scan is composed of 1D signals in the z-direction (A-scans in Fig. 1b). Manual analysis of this data is tedious, time-consuming, and prone to error. Over the past two decades, researches on OCT image processing has been devoted to the main areas: segmentation of the retinal layers [1, 2, 4–9], classification [10, 11], enhancement, and denoising [12–14].

Today, deep learning algorithms are widely used in this application and their performance is in presence of abnormalities [5, 15–22]. Even though a great number of OCT data is available online, they may not necessarily resemble specific datasets. Namely, if the network is trained on less similar online datasets, the difference in device-specific parameters, size, resolution, noise, etc., will affect the performance on local datasets. Furthermore, most of the online datasets only contain the label for each image and delineation of the boundaries is rarely given. To deal with mentioned limitations, the proposed method produces the new OCT data with a similar appearance to limited local datasets. Furthermore, the synthetic OCTs include the boundary locations for each scan, which is appropriate in applications like segmentation.

On the other hand, the proposed synthetic data can be used in the evaluation of segmentation methods to measure the robustness to noise and the performance in presence of abnormalities. Denoising methods can also be evaluated in different noise levels, on abnormal structures, and in 3D data.

In previous works, synthetic images are developed as a benchmark to compare and evaluate the performance of medical image processing algorithms such as CT scans [23, 24], MRI [25], and ultrasound images [26]. In recent years, synthetic retinal fundus images [27–29], as well as OCT retinal images have been proposed [30–33]. Serranho [31] proposed a mathematical formulation to produce synthetic OCT boundaries on 10 manually segmented healthy human OCT data. Shahrian [30] improved Serranho’s method by dividing the retinal region into small blocks and fitting simple basic functions to the boundaries. They produced synthetic data in the presence of cysts but blood vessels are not considered. In [33] synthetic images were generated using the statistical feature of 14 real ocular images of different animals. The initial segmentation was done manually for 7 retinal layers and a statistical model was used to produce synthetic images. In [32] two retinal layers (ILM, RPE) were extracted and the thickness profile was calculated for the distance between these two regions. By classifying A-scans with the same thickness values, the appearance model for each position and thickness was estimated. A mathematical model was constructed from the thickness maps and the appearance model was used to select the corresponding A-scans. In our primary work [34], synthetic data was constructed using 2D Active Shape Model (ASM) [35–37] to construct boundaries and the corresponding point distribution model (PDM) in normal OCTs. Generative adversarial networks (GANs) [38–41] can be considered as the main competitor of the proposed method. However, such methods are good candidates for synthetic data in classification problems. Namely, they produce lots of OCTs with different diseases by utilizing normal OCTs, with aim of compensating the limited number of abnormal data. However, the annotations are usually missed in these methods, making them improper for segmentation tasks. The proposed method conquers GAN methods by producing the image and the labels (for retinal boundaries and the abnormalities) in a single pipeline.

Most of the previous methods do not utilize the information in the third dimension of the data and are concentrated on mathematical formulas to shape the OCT curves in 2D. In this work, we propose to generate OCT volumetric data by using 3D ASM [42, 43]. The method is therefore applicable in 3D deep learning methods and explicitly includes prior knowledge about the volumetric data. Furthermore, we focused on the synthesis of data with abnormalities in one of the most complicated eye diseases, diabetic macular edema (DME) [44].

The rest of this paper is organized as follows: In Section II, we describe two proposed methods for generating the synthetic data in a 3D format and in presence of DME. In Section III the result and experiments are reported. Finally, in Section IV we discuss the proposed method and give concluding remarks and hints for future works.

## Materials and methods

**Fig. 2:**
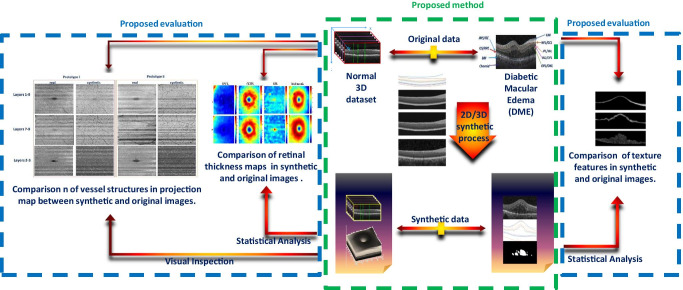
Block diagram of the proposed method and the evaluations

The block diagram of the proposed method is demonstrated in Fig. 2. The background theory is similar to our primary work (for more detailed information please refer to [34]).

### Proposed 3D model

#### Three-dimensional OCT dataset

Two different datasets are used in this work. The first dataset is obtained from Spectralis SD-OCT Heidelberg Eye Explorer (HEYEX) version 5.1 (Heidelberg Engineering, Heidelberg, Germany)*;* the study was approved by the regional bioethics committee in Isfahan University of Medical Sciences under ethics ID: IR.MUI.MED.REC.1398.647[45].The size of each data is 19 × 512 × 496 voxels, where 19 is the number of cross-sectional images in each data. Five 3D data from the healthy population are used in the training.

The second dataset is acquired from a Topcon device with a size of 512 × 650 × 128 (dense B-scans) [46] (available in https://misp.mui.ac.ir/fa/node/1388) to prove the effectiveness of the method in different devices and to produce projection images representing the vessel structures. The study was approved by the regional bioethics committee in Isfahan University of Medical Sciences under ethics ID: IR.MUI.MED.REC.1398.697.

#### Synthetic boundaries using 3D Active Shape Model (3D-ASM)

Originally developed ASM by Cootes [47] is expanded to 3D in [43]. ASM allows arbitrary shapes to be modeled using an adaptive shape technique even when deformed [48]. The main advantage of a 3D model is that it explicitly includes prior knowledge about the volumetric data and learns the mean and variances of the 3D object using Principal Component Analysis (PCA). In the 3D version, PDM contains x, y, and z position and ASM sets up a trained deformed model to a set of identifiable points in the image. The correspondence of the points should be essentially considered. Fortunately, the retinal layers have a relative order, and this makes the point correspondence less complicated. Namely, the ASM model includes a series of curves that span from left to right of the image. Accordingly, the numbering of each boundary starts with point 0 to the left-most pixel, point 26 to the middle pixel (located on fovea in macular regions of the normal B-scans), and point 51 to the right-most pixel. we distributed the middle points, evenly. A 3D alignment is then applied on the point cloud to put OCTs into similar rotation, translation, and size. To generate a PDM, the boundary information in each OCT data is described using a set of $n$ sample points:

1 $${x}_{i}={\left({x}^{1}, {y}^{1}, {z}^{1},\dots ,{x}^{n}, {y}^{n}, {z}^{n} \right)}^{T}$$

where ${x}_{i} (i=1,..,N)$ represents each 3D OCT boundary in the training set. The mean vector $\overline{x }$ and covariance matrix $S$ is then computed for all training data cloud:

2 $$S=\frac1N{\textstyle\sum_{i=1}^N}(\overline x-x_i){(\overline x-x_i)}^T$$

PCA is then performed on covariance matrix to calculate the eigenvector $({\mathrm{p}}_{\mathrm{i}})$ and eigenvalues $({\upsigma }_{\mathrm{i}})$ of the training set. Depending on the amount of variation in the training set (represented by the model), the eigenvector corresponding to t largest eigenvalues is selected to form$\mathrm{p }=({\mathrm{p}}_{1} , {\mathrm{p}}_{2} ,\dots {\mathrm{p}}_{\mathrm{t}})$.

Considering the covariance matrix, the most important modes of changes in the point distribution of the retinal layers can be modeled using a limited number of eigenvectors, corresponding to the large eigenvalues (principal component variances). To select good-enough eigenvectors and to eliminate the less important ones from the calculations, the percentage of total variance explained by each principal component is considered and components with a percentage more than 0.94 were chosen (The selected value for t in this study is 5).

Each OCT point cloud can be approximated by:

3 $$X\cong \overline{x }+P*b$$

where $b$ is a $\mathrm{t}$ -dimensional vector containing model parameters. By changing these parameters, different samples of the OCT point cloud can be created as synthetic data. Assuming Gaussian distribution for training data, only limited values of parameters $b$ will lead to reliable synthetic OCT data (with enough similarity to the original set). To apply this limitation, the variance of parameter ${b}_{i}$ for samples in the training set is given by ${\upsigma }_{i}$ and newly assigned ${b}_{i}$ s should be limited to:

4 $$-3\sqrt{{\upsigma }_{i}}\le { b}_{i }\le + 3\sqrt{{\upsigma }_{i}}$$

To generate the model, five healthy volumetric OCT data are used in the training stage. Since ASM requires a set of points for training, the location of the boundaries in the training data must be extracted first, and important points must be given to the ASM as a matrix of points. For extraction of the boundaries, a diffusion map algorithm is used [4] and then the extracted boundaries are modified under the supervision of an expert by the semi-automatic method [49] to fix any undesirable boundary. These boundaries are fed as input to the proposed 3D ASM. Figure 1. c shows the segmented boundaries on a sample OCT B-scan and corresponding retinal layers are as follows: Inner Limiting Membrane (ILM), Nerve Fiber Layer (NFL), Inner Plexiform Layer (IPL), Inner Nuclear Layer (INL), Outer Plexiform Layer (OPL), External Limiting Membrane (ELM), Outer Segment Layer (OSL), Retinal Pigment Epithelium (RPE), and Bruch Membrane (BM).

**Fig. 3:**
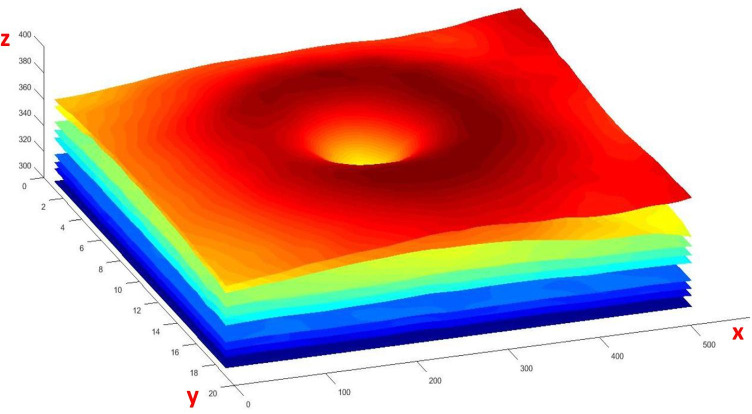
An example of 3D OCT boundaries with nine synthetic surfaces visualized in 3D. The x, y, z-axis in this picture correspond to the dimensions of the 3D data [512 × 19 × 496]

Fifty-one points were selected for each boundary in each B-scan (969 Points for each boundary in each 3D volume) and after constructing the 3D PDM, data synthesis is done by changing the parameters in a limited range (Eq. 4). A sample of 3D synthetic boundaries with nine synthetic surfaces is demonstrated in Fig. 3.

#### Synthetic layer content in 3D

After constructing 3D boundaries for each data, the average brightness of each layer for a set of B-scans is selected as the brightness of the newly constructed layer in synthetic data. For the production of each new data, a fraction of the training dataset is considered for brightness transfer to provide the diversity of brightness expected in the synthetic data.

For the production of synthesized vessels, another ASM model is designed according to the intensity profiles of vessels in the training set. The local brightness of the location hosting the vessel is considered and each new vessel profile is normalized to match the host region [34]. The vessel intensity profiles are then placed in locations calculated using the shadow-based method [50].

Speckle noise is then applied with different standard deviations to make 3D data with a variety of noise levels. The noise level is not a fixed value in our synthetic model. Instead, we construct many synthetic images with a wide range of noise levels (similar to what we observe in real data). This collection of noise levels can truly represent a real database. Furthermore, one can choose to have a high level of noise (to test the robustness of a segmentation method). Or, one may use noiseless and highly noisy data together to measure the performance of a denoising method.

Finally, to create complete synthetic data, the choroid region and the background of a reference 3D image are added to the generated 3D data. Each synthesized 3D data is finally accompanied by the location of retinal boundaries and adding this delineated dataset to the training set of deep learning methods is expected to increase the performance of the network as a tailored augmentation method.

### Proposed model in presence of abnormality

**Fig. 4:**
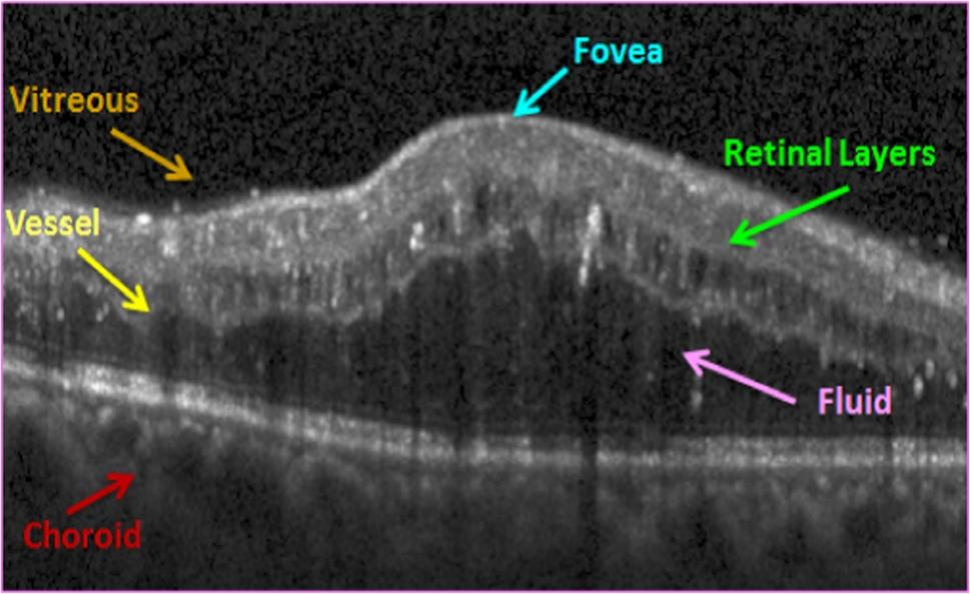
Sample OCT image for an eye with DME

DME is one of the major causes of blindness. About one fifth of patients with diabetes for a long time, suffer from DME. Its pathological features are mainly manifested in fluid accumulation in the retina [51]. Figure 4 shows a sample of OCT images with DME. Several methods have been proposed for the segmentation of the OCT with DME [52–54]. The new deep learning methods seem to be promising in this complicated segmentation, but the limited number of available delineated ground truth and the high burden of manual segmentation for making gold standards can be considered as the main problems in applying deep learning methods to solve this problem.

#### OCT dataset with DME

**Fig. 5:**
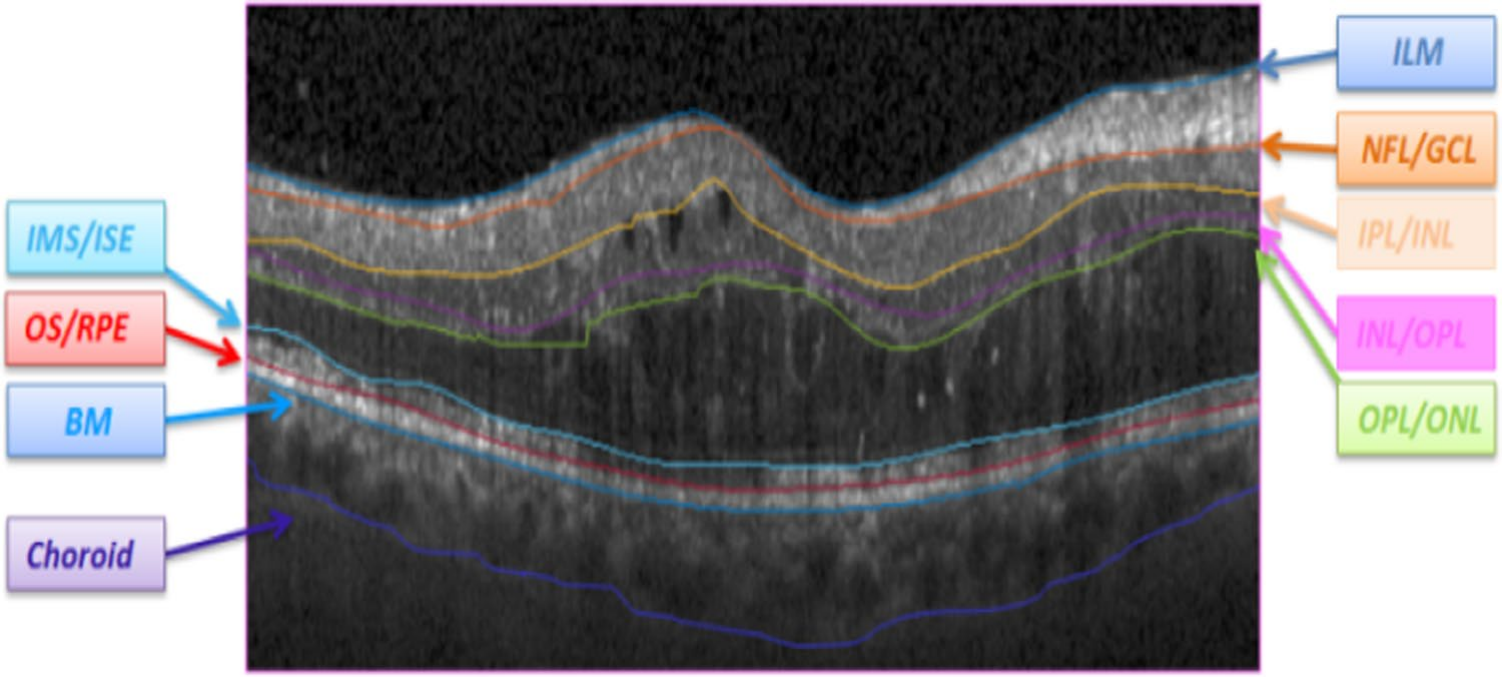
Boundaries of retinal layers in presence of abnormalities (DME) [55]

To train the proposed model, the dataset by Chiu et.al [55] is selected due to providing both locations of retinal boundaries and the fluids in each B-scan. The dataset contains ten OCT volumes (110 B-scans). Each B-scan is associated with delineated boundaries (Fig. 5).

Thirty-five B-scans with all eight boundaries are selected from this dataset. Since the thickness of the choroidal layer is important in DME, the 9th boundary is also segmented manually in the images of the training set and is used in synthetic OCTs.

#### Synthetic boundaries using 2D ASM on DME data

Two-dimensional ASM [34] is utilized for the synthesis of DME boundaries. The overall strategy is a 2D version of what was described in the previous section. The only difference is that due to the presence of DME, overshoots in boundaries of this dataset go beyond the limited range of parameters in Eq. (4). Therefore, this limitation is eliminated.

#### Synthetic layer content

To mimic the texture, intensity variations, and blood vessels in synthetic data, the layer contents are transferred, resized, and replaced column-wise from most similar OCT data in the training set. The similarity is determined based on the shape of the first boundary [34]. The presence of retinal fluids in DME data is an important issue that should be considered individually [56–58]. To address this issue, the fluids are also transferred column-wise from the training dataset. Each synthesized data from the DME dataset is therefore accompanied by the location of boundaries plus the location of fluids. Such a delineated dataset can then be used in deep learning methods as an augmented set of data for training.

## Result

### 3D synthetic data

**Fig. 6:**
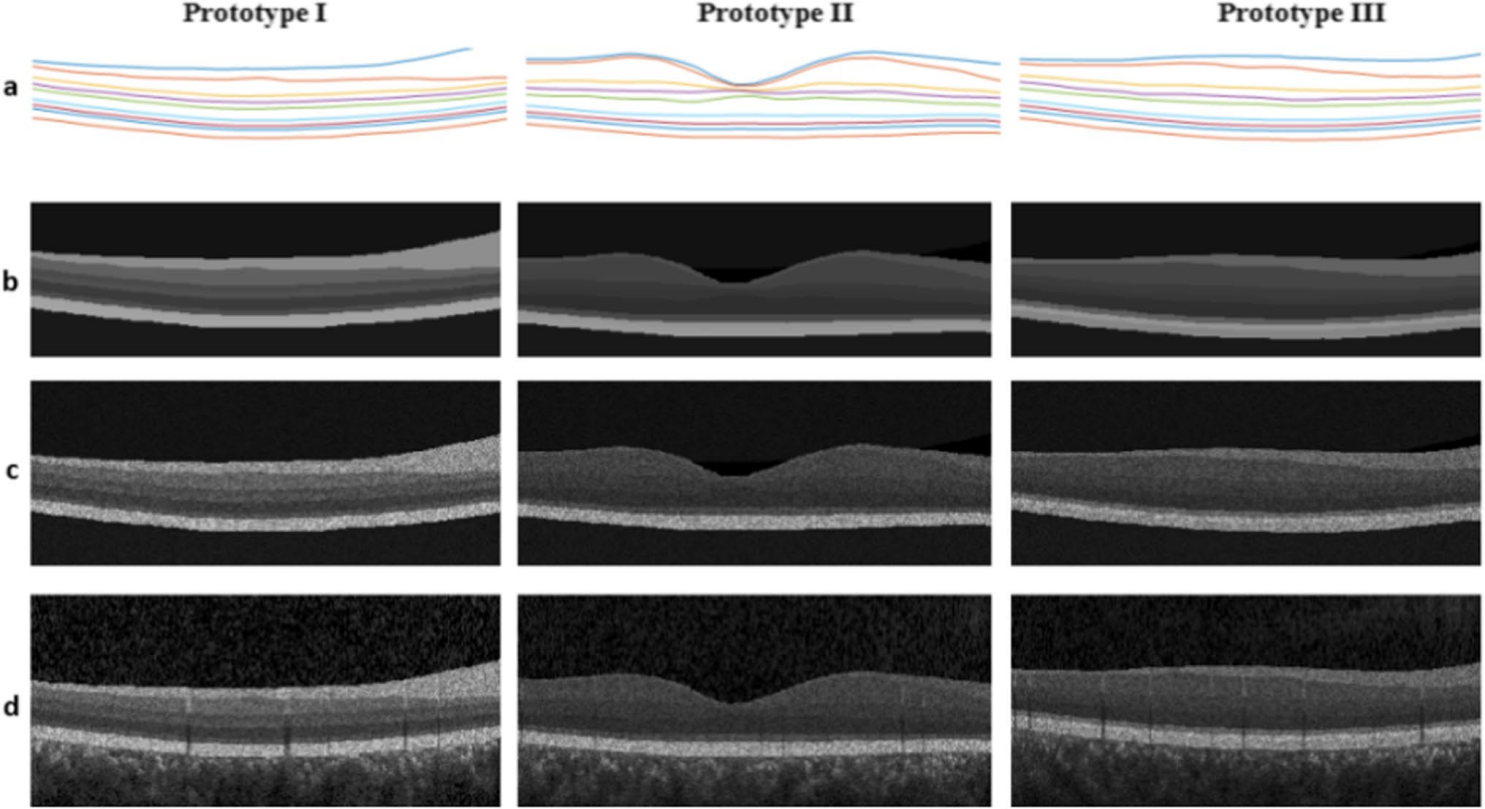
Different stages of Synthetic OCT data for 3 prototypes, a) synthetic boundaries, b) Noise-free synthetic image, c) Adding noise to the synthetic image, d) Adding synthetic blood vessels

Figure 6 demonstrates the whole process for 3D sample data, where the columns are 2D example B-scans and the rows correspond to different stages of the algorithm. The first row shows the synthetic boundaries. The second row is about adding the proper brightness as layer content. The third row shows the process of adding speckle noise, and the fourth row contains the final created images with background, choroid region, and the blood vessels. It is also important to discuss the prevalent problem regarding crossing boundaries. Since the training boundaries were securitized carefully to avoid any crossing, the synthesized boundaries are without crossing. It is important to note that in the proposed method, all boundaries make a single ASM model, and accordingly the model can learn the relative location of the boundaries, rather than only learning the single individual curves, which may interfere finally*.*

### DME synthetic data

**Fig. 7:**
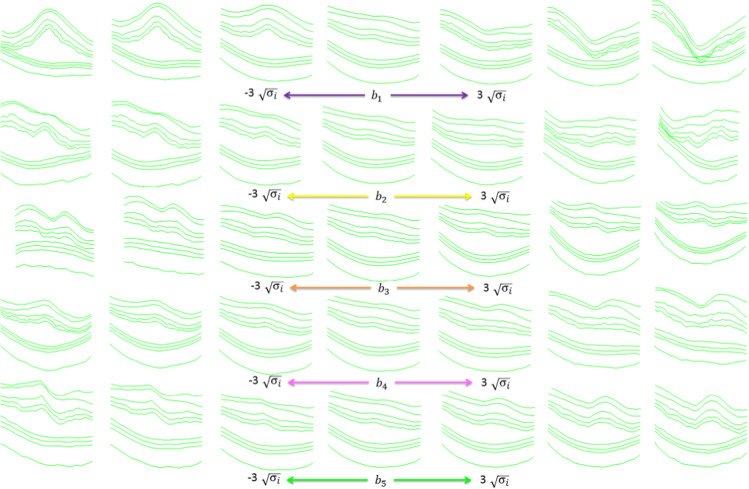
Effect of varying first five parameters of the model. Each row is obtained with different values of parameters and leads to new synthetic data

To synthesize abnormal data, the ${b}_{i}$ values can change the appearance of the data and lead to new datasets. Figure 7 shows this effect in detail and each row is created by changing the value of each ${\mathrm{b}}_{\mathrm{i}}$ (${\mathrm{b}}_{1}$ in the first row, ${\mathrm{b}}_{2}$ in the second row, …), following Eq. 4. The value of $3\sqrt{{\upsigma }_{\mathrm{i}}}$ is designed to guarantee the outlier data will not be produced.

**Fig. 8:**
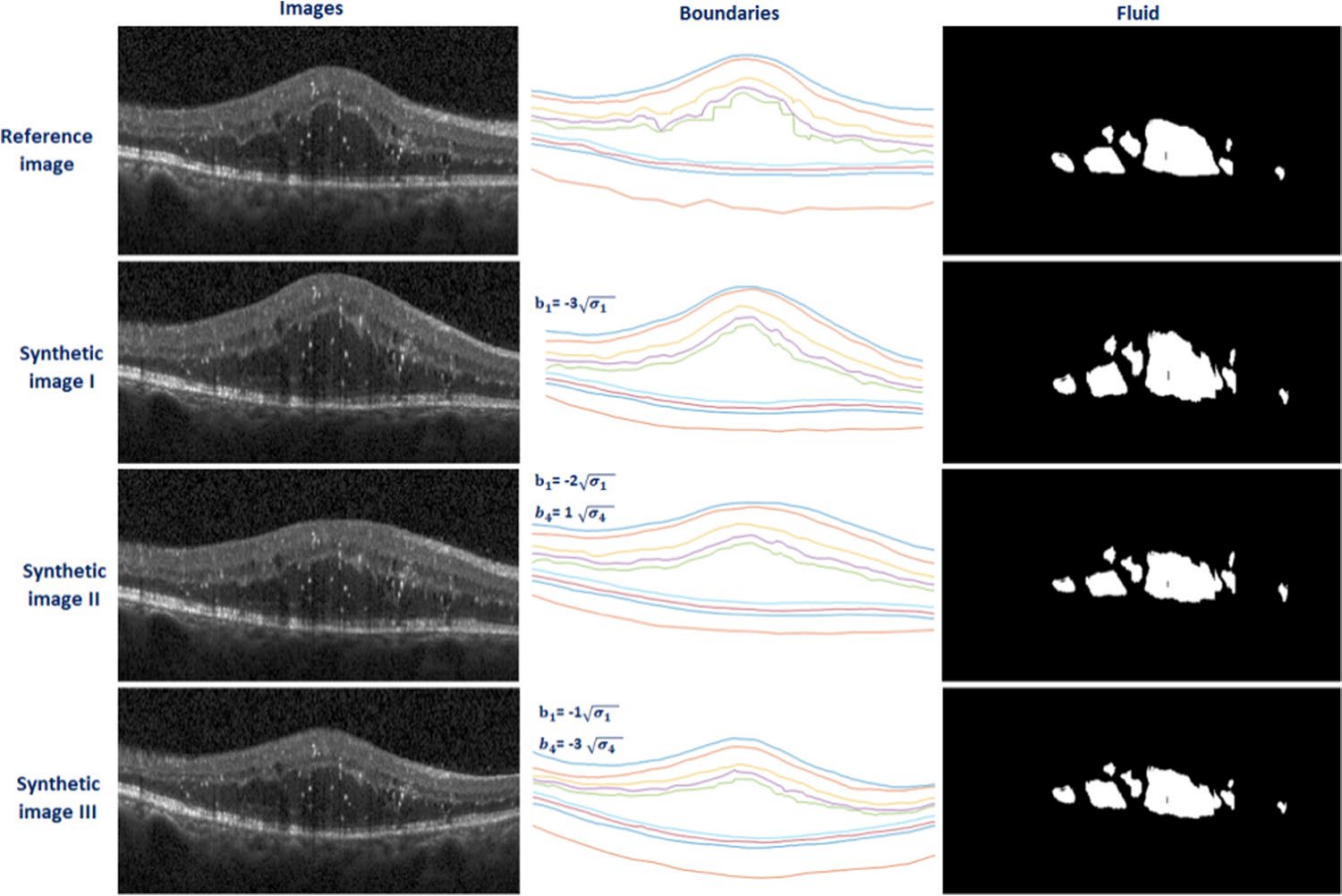
Examples of synthetic DME images obtained from a single reference image with varying ${\mathrm{b}}_{\mathrm{i}}$ parameters in rows 2 to 4. The corresponding boundaries and fluid locations are shown in the second and third columns

Each different combination of ${\mathrm{b}}_{\mathrm{i }}\mathrm{s}$ is expected to produce new OCT data. To demonstrate this fact, examples of synthetic DME images obtained from a single reference image with varying ${\mathrm{b}}_{\mathrm{i}}$ parameters are shown in Fig. 8. The first row shows the reference image with its fluid mask and the boundaries. The 2nd to 4^th^ rows show three samples of synthetic images with their corresponding ${\mathrm{b}}_{\mathrm{i}}$ parameters, boundaries, and fluid masks.

**Fig. 9:**
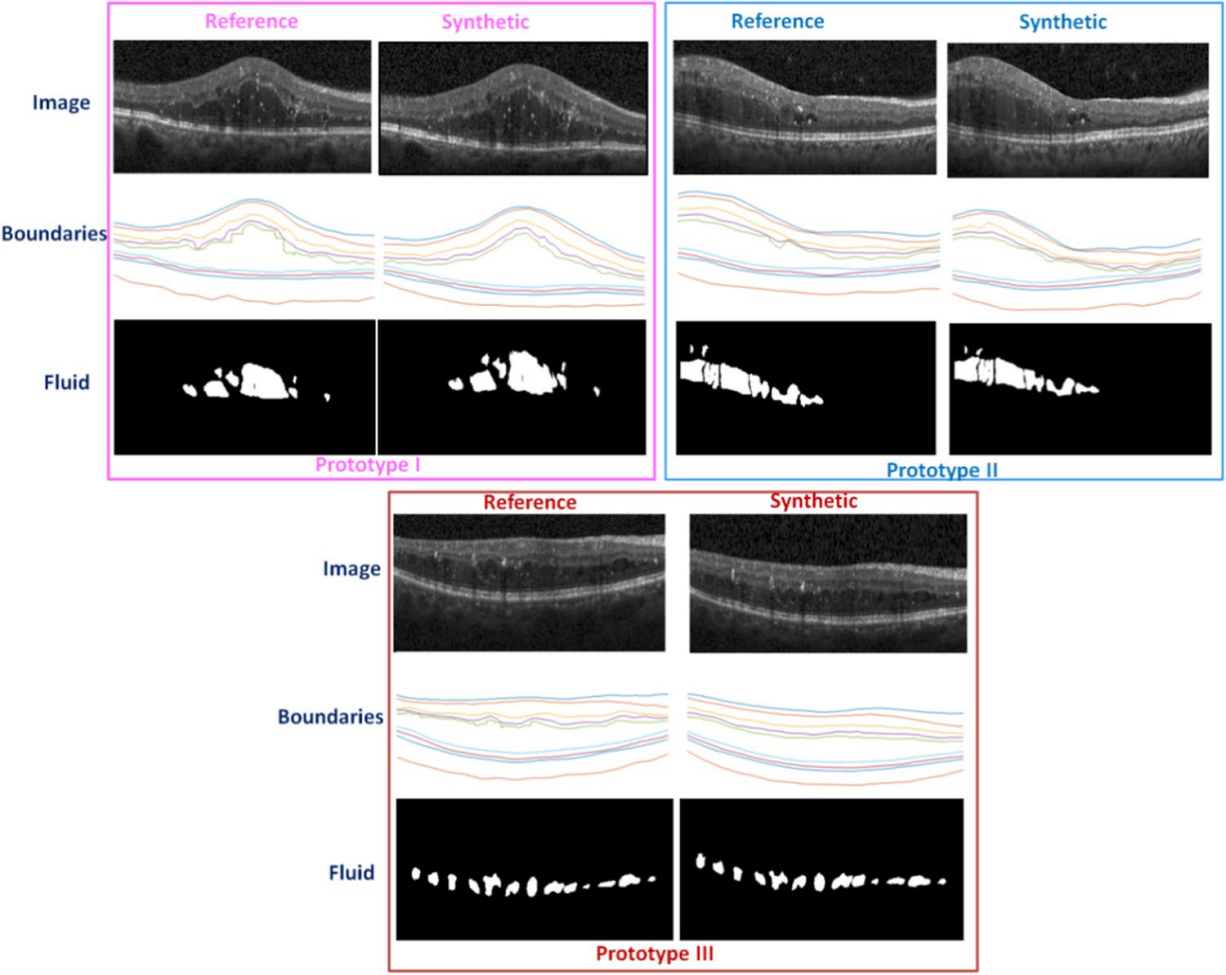
Three different reference images and one sample of synthetic data for each

Figure 8 was developed based on a single reference image and discussed variations of synthetic data. In Fig. 9, however, we aim to show the performance of the proposed method for different reference images. Figure 9 demonstrates three different reference images and one sample of synthetic data for each. Therefore, three different reference images are selected and one specific synthesized image (out of many possibilities) is shown along. The boundaries and fluid specifications for both reference and synthesized images are presented to show similarities and differences among them. It can be seen that the boundaries and fluids of the synthetic images do not exactly match the reference image but are similar. This allows us to create a large number of images leading to significant augmented data from the real dataset to be used in the training of a deep learning algorithm.

### Statistical analysis and evaluation of the results

**Fig. 10:**
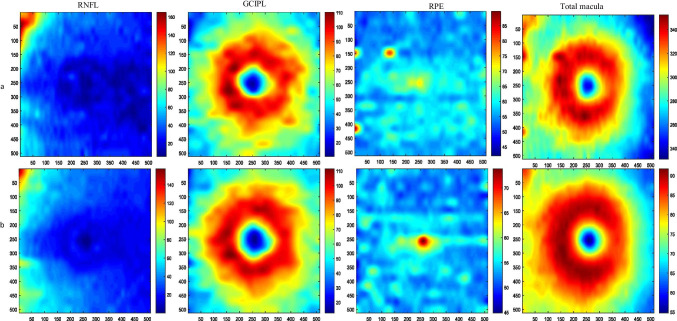
Retinal thickness maps of synthetic and original images a) Thickness maps of reference data (RNFL, GCIP, RPE, and total macula), b) Thickness maps of one sample synthetic data

To show that synthetic images with the proposed method are statistically similar to the original datasets, we used a normal population of five 3D OCT data as reference images and synthesized 25 new 3D OCT data. It is expected that the synthesized dataset would have a similar anatomical structure to the original ones. For this purpose, one of the main characteristics of OCT images (thickness maps of important retinal layers) is considered. Values of thickness maps from retinal layers play an important role in diagnosing and monitoring layer thickness changes in retinal diseases [59]. By differentiating the location of two successive boundaries, a thickness map of the corresponding layer can be obtained. The macular thickness map is calculated for the whole retinal thickness and important individual layers including RNFL, GCIPL, and RPE are calculated. Figure 10 shows the thickness maps of mentioned retinal layers and the total retina for one sample synthetic and the related reference data. The numerical values of thickness maps are compared between the original and synthesized data and no statistical difference (*p*-value > 0.05) was observed between both groups, which mean that the synthetic dataset can be used as a statistically acceptable representative of the original dataset (Table 1).

**Table 1:** Comparison of thickness maps in important retinal layers among real and synthesized groups

	Total macula	RNFL	GCIPL	RPE
Real Normal Data (mean ± SD)	310.90 ± 12.80	29.72 ± 3.10	76.67 ± 2.89	55.55 ± 3.23
Synthetic Normal Data(mean ± SD)	308.07 ± 5.013	29.84 ± 1.92	75.86 ± 2.21	54.98 ± 1.2
*P* value	0.8432	0.4353	0.8617	0.7904

**Fig. 11:**
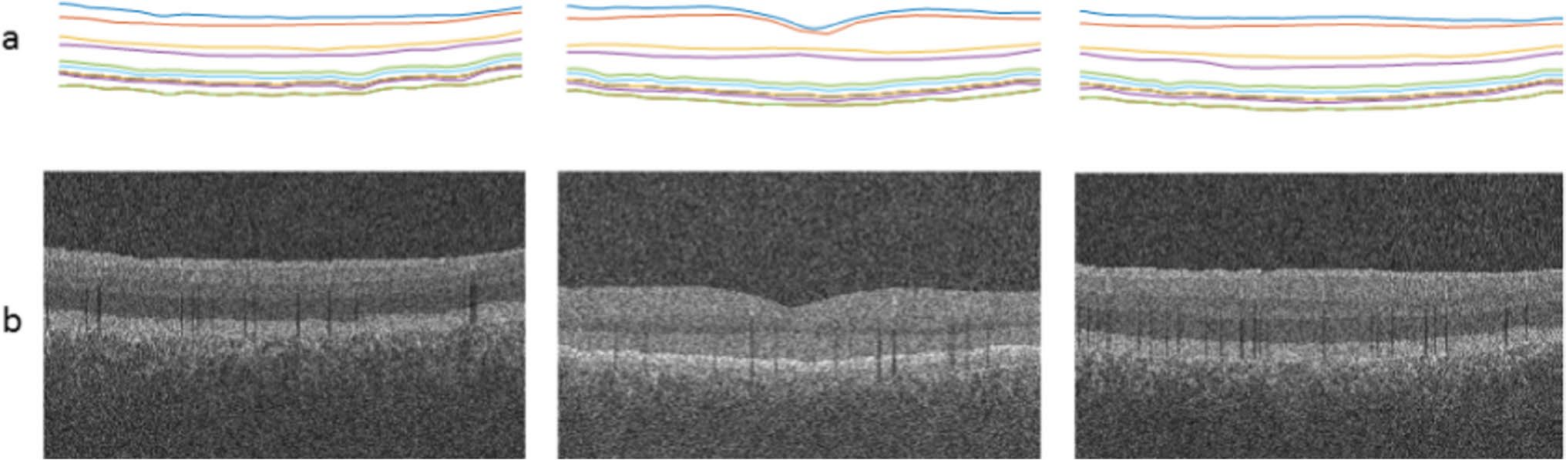
Synthetic OCT data for 3 prototypes, a) synthetic boundaries, b) synthetic images with vessel shadows in different locations

**Fig. 12:**
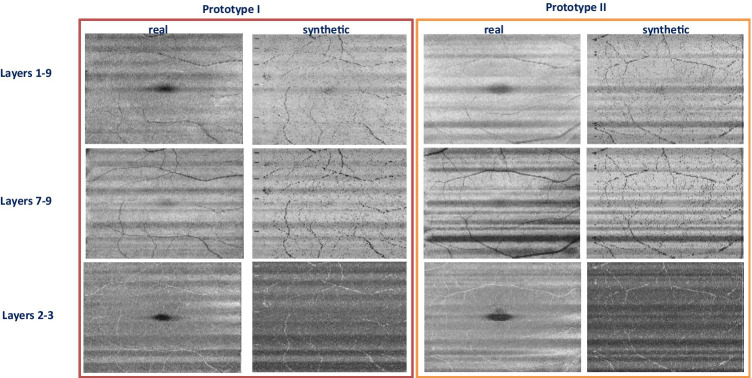
Projection images from 3D OCT data by calculating the mean value of brightness in the whole retina (first row), in outer layers (second row), and in inner layers (third row). Different couples of retinal layers (Fig. 12, first row). Two prototype examples are presented in right and left panel and in each case; the columns corresponding to synthetic and real data are demonstrated to show the similarity of the synthesized vessels

Another important information in proposed synthetic data is the presentation of the vessels. The second dataset is from a Topcon device with dense B-scans [46] is used to produce projection images representing the vessel structures (it is not practical to make projections in sparse datasets). Figure 11 shows three examples of synthetic data with corresponding boundaries. Each synthetic B-scan carries vessel-related information, similar to real B-scans. Each vessel leads to bright shadows in upper boundaries (inner retinal layers) and dark shadows in lower regions (outer retinal layers). To evaluate the correct correspondence between the synthetic vessel and the real data, 3D synthetic OCTs are considered. Projection images are constructed by calculating the mean value of brightness between different couples of retinal layers (Fig. 12, first row). By constructing projection images from outer retinal layers, the vessels are presented in dark mode (Fig. 12, second row) and the similar projects from inner retinal layers yield to the bright presentation of the vessels (Fig. 12, third row). Visual comparison of such projections in two prototype examples, shows the acceptable performance of the synthetic data in the creation of vessels compared to real data (Fig. 12, real and synthetic columns).

**Fig. 13:**
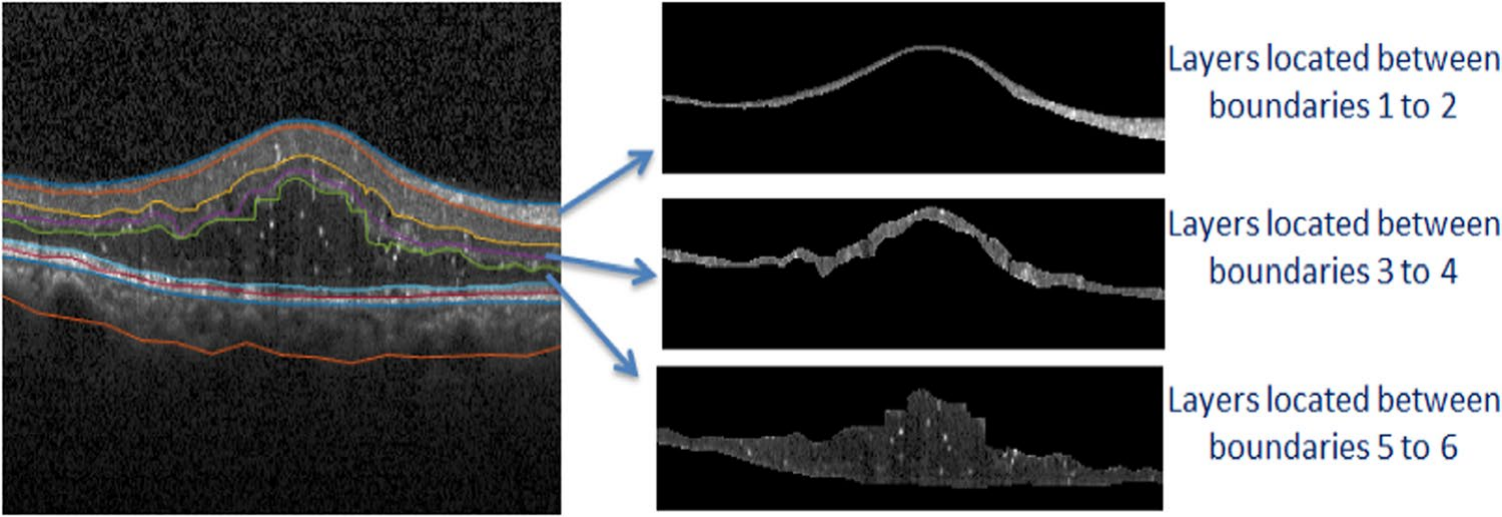
DME image with selected layers for feature extraction

The other crucial information in synthetic OCTs is texture features. To show that these features are preserved in synthetic data, texture-related features are calculated in similar regions from synthetic and real data. The selected features that measure the spatial relationship of the pixels are the gray-level co-occurrence matrix (GLCM). The GLCM functions determine the texture by calculating how often pairs of the pixel with specific values and in a specified spatial relationship occur in an image, by creating a co-occurrence matrix, and then extracting statistical measures from this matrix. As an example for three different regions (in layer 1(between boundaries 1 and 2), layer 3 (between boundaries 3 and 4), and layer 5 (between boundaries 5 and 6), elaborated in Fig. 13), four texture features (shown in Eqs. 5–8) are extracted. The first feature, Energy, provides the sum of squared elements in the GLCM[60]:

5 $$\mathrm{Energy}={\textstyle\sum_{i=1}^N}{\textstyle\sum_{j=1}^N}{p(i,j)}^2$$

where $p(i,j)$ is generated by counting the number of times a pixel with the value *i* is adjacent to a pixel with value *j* and then dividing the entire matrix by the total number of such comparisons. Each entry is therefore considered to be the probability that a pixel with the value *i* will be found adjacent to a pixel of value *j*. Contrast returns a measure of the intensity contrast between a pixel and its neighbor over the whole image:

6 $$Contrast={\textstyle\sum_{i=1}^N}{\textstyle\sum_{j=1}^N}\left(i-j\right)^2p(i,j)$$

Homogeneity Measures the closeness of the distribution of elements in the GLCM to the diagonal:

7 $$\mathrm{Homogeneity}={\textstyle\sum_{i=1}^N}{\textstyle\sum_{j=1}^N}\frac{p(i,j)}{1+\left(i-j\right)^2}$$

Correlation Returns a measure of how correlated a pixel is to its neighbor over the whole image:

8 $$\mathrm{Correlation}={\textstyle\sum_{i=1}^N}{\textstyle\sum_{j=1}^N}\left(\frac{i-\mu_x}{\sigma_x}\right)\left(\frac{j-\mu_y}{\sigma_y}\right)p\left(i,j\right)$$

**Fig. 14:**
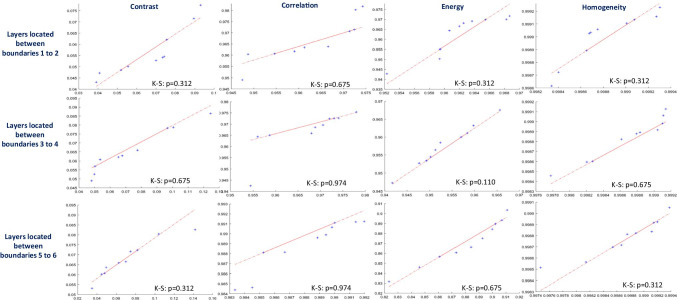
Q-Q plots to compare the similarity of distributions for calculated texture features (columns) in 3 selected regions (rows) on 10 real and 10 synthetic images. Each plot is enriched by *p*-values of the Kolmogorov–Smirnov test (K-S) supporting the difference between sample distributions when the value is < 0.05 (in case of level of significance *α* = 0.05)

To provide a comparison of distributions of extracted features between synthetic and real data, the quantile–quantile plot (Q-Q plot)[61] is used. In the case of the same distribution in synthetic and real data, the points are expected to be along a straight line with a slope of 1, while the deviation of the points from this line shows the difference in distribution. Since the Q-Q diagram is only a visual technique for comparing data, this method can be combined with the Kolmogorov–Smirnov statistical test [61]. As it can be seen in Fig. 14, even in cases that the points have slightly digressed from the straight line, the *p*-values of the Kolmogorov–Smirnov test rejected the null hypothesis and showed the same distribution in texture features of the real and synthetic data.

## Discussion and conclusion

The proposed model is designed to fill the available gap in synthetic OCT data by employing important features like intensity, blood vessels, global noise, and texture information in the synthesis of the new data. The proposed method is applicable to 3D data and can work in presence of abnormalities, the features that have not been explored before. In previous studies [30–32], only three layers of the retina are extracted and synthetic images are made based on this low dimension of the input data. The proposed method with nine layers is designed to solve this problem. Furthermore, to the best of our knowledge, it is the first time that the choroid region is also labeled and used in the constructed data. Additionally, in previous studies, the number of generated data is limited and the labeled data set is not freely available.

**Fig. 15:**
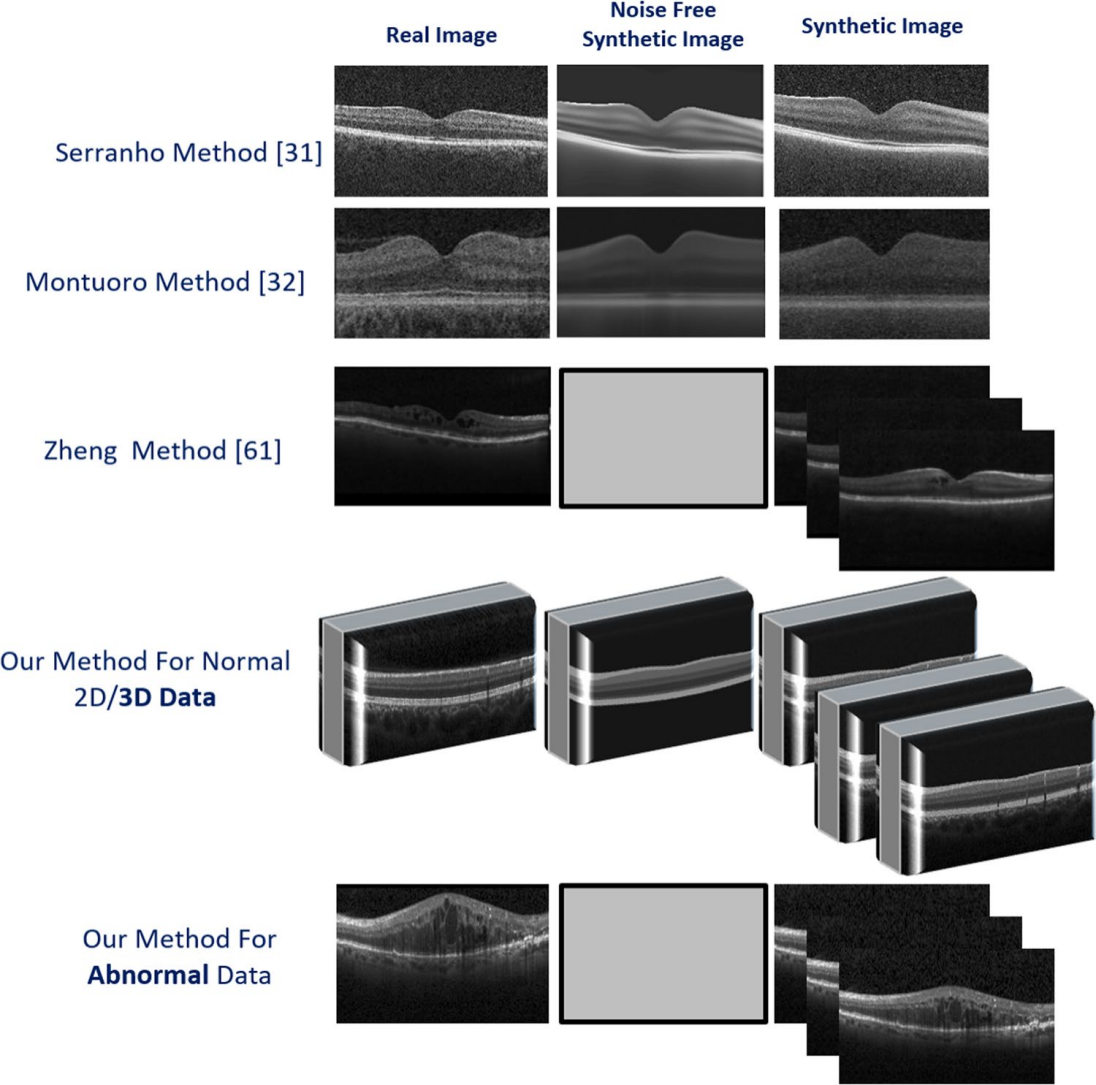
Different synthetic OCT methods are compared with proposed models. The first and second rows show methods with the ability to make noise-free images, similar to the proposed method. The third and fourth rows show the similarity of GAN methods to our method in producing multiple images from limited data. The ability of the proposed method in the production of 3D data and abnormal cases is also shown in the fourth and fifth rows

To provide a visual comparison with some previous methods, Fig. 15 shows the synthetic data samples from previous works in comparison with the proposed method. This shows characteristics like noise-free version, the possibility of making 3D data, and production of multiple images from a single original image, elaborated in the next paragraphs. As it can be seen in Fig. 15, Serranho [31] and Montuoro [32] produced 2D OCTs and noise-free data. However, both of them lack some important information. For instance, Serranho’s [31] method ignores the presence of the vessels and the number of the modeled layers are very limited in both of them. The choroidal region is also ignored in both. Furthermore, both methods preserve the location of the boundaries from the original image and new boundary locations are not constructed..

An important aspect of the synthetic algorithms is their ability to synthesize a high number of new images and this is considered in our proposed method and GAN models like Zheng [40]. In both algorithms, the model receives a limited set of OCTs and makes an almost unlimited number of synthetic data.

On the other hand, previous methods are developed for normal OCTs and have ignored complicated cases with diseases. In this paper, we elaborated the proposed method to work perfectly with DME cases, as one of the most complicated diseases, and the synthetic data with boundaries and fluid locations are then constructed. In our previous work [34], synthetic data was fabricated using 2D data. The proposed 3D construction is also novel in this work and provides the opportunity for 3D data augmentation which is of real importance in deep learning applications.

The innovations of this method can therefore be summarized as below:

1. Synthesis of 3D data
  In 2D OCT data, one cross-section can be demonstrated which can never represent the whole structure of the eye. Therefore, 2D data cannot be used in the evaluation of the diseases which affect the whole eye. Even, if a large amount of individual 2D data is generated for successive positions, the inter-correlation between them would be lost and they cannot be considered as a 3D representation. With the proposed method, 3D data can be synthesized and the 3D structure of the layers is expected to maintain.
2. Production of synthetic data in presence of abnormalities
  Today, deep learning algorithms are used to process OCT images in presence of abnormalities, which requires a large number of labeled data in the training step. A low amount of labeled data in presence of abnormalities is the main limitation in deep learning methods and this problem can be solved by producing synthetic data with the proposed method.
3. New method of creating image textures

Instead of using the complicated steps of previous works [34], we proposed a simple texture transfer method applicable to abnormal OCTs. The textural information of the fluids according to reference data is therefore guaranteed to remain.

The proposed method is also suffering from some limitations. As discussed above, the proposed method can preserve crucial information regarding the location of boundaries, the vessels, and the texture. However, each of the mentioned parameters may not be a faithful representation in all cases. We can assure that the proposed method is a true augmentation regarding the generated boundaries and any machine learning algorithm with emphasis on the boundaries can use the augmented data correctly. The vessels, as elaborated before, are visually assessed to have acceptable similarity with the original data, however, a very detailed match can not be guaranteed. Since many papers in the literature extract information using texture analysis, the proposed method for abnormal data can preserve the full texture information by acquiring the texture from the most similar image in the training data. However, our older method [34] may fail in such cases since the mean brightness of each layer accompanied with speckle noise may not essentially be an ideal model for the texture of the retinal layer.

As another limitation, due to the high computational load of the method in presence of densely selected points for ASM, the proposed 3D method is using only a limited sparse number of landmark points. Therefore, if some diseases would lead to small changes in the retina, the location of the abnormality may be missed with our sparse sampling. Furthermore, the proposed method relies on the exact location of the annotated points in the training step. Therefore, it is crucial to have a very precise segmentation of boundaries and the abnormalities by an expert. Any mistakes would affect the proposed method.

In conclusion, the proposed method fills the gap in previous OCT synthetic methods by addressing complicated issues like 3D representation and presence of the abnormalities. Despite undeniable limitations, the method is shown to be capable of modeling the boundaries, vessels, and texture of the OCT data and be an acceptable augmentation method for future methods needing a high number of the labeled OCT data.

## References

[1] Ben-Cohen A, Mark D, Kovler I, Zur D, Barak A, Iglicki M, Soferman R (2017) Retinal layers segmentation using fully convolutional network in OCT images. RSIP Vision, 1–8

[2] Schmitt JM (1999). Optical coherence tomography (OCT): a review selected topics in Quantum Electronics. , IEEE Journal of.

[3] Hassenstein A, Meyer CH (2009). Clinical use and research applications of Heidelberg retinal angiography and spectral-domain optical coherence tomography–a review. Clin Experiment Ophthalmol.

[4] Kafieh R, Rabbani H, Abramoff MD, Sonka M (2013). Intra-retinal layer segmentation of 3D optical coherence tomography using coarse grained diffusion map. Med Image Anal.

[5] Fang L, Cunefare D, Wang C, Guymer RH, Li S, Farsiu S (2017). Automatic segmentation of nine retinal layer boundaries in OCT images of non-exudative AMD patients using deep learning and graph search. Biomed Opt Express.

[6] Danesh H, Kafieh R, Rabbani H, Hajizadeh F (2014). Segmentation of choroidal boundary in enhanced depth imaging OCTs using a multiresolution texture based modeling in graph cuts. Comput Math Methods Med.

[7] Gao Z, Bu W, Zheng Y, Wu X (2017). Automated layer segmentation of macular OCT images via graph-based SLIC superpixels and manifold ranking approach. Comput Med Imaging Graph.

[8] Dodo BI, Li Y, Eltayef K, Liu X (2019). Automatic annotation of retinal layers in optical coherence tomography images. J Med Syst.

[9] Gonzalez-Lopez A, Ortega M, Penedo MG, Charlon P (2015). A web-based framework for anatomical assessment of the retina using OCT. Biosys Eng.

[10] Abdolmanafi A, Duong L, Dahdah N, Cheriet F (2017). Deep feature learning for automatic tissue classification of coronary artery using optical coherence tomography. Biomed Opt Express.

[11] Mousavi E, Kafieh R, Rabbani H (2020). Classification of dry age-related macular degeneration and diabetic macular oedema from optical coherence tomography images using dictionary learning. IET Image Proc.

[12] Apostolopoulos S, Salas J, Ordóñez JL, Tan SS, Ciller C, Ebneter A, Zinkernagel M, Sznitman R (2020). Automatically enhanced oct Scans of the Retina: A proof of concept study. Sci Rep.

[13] Chen Z, Zeng Z, Shen H, Zheng X, Dai P, Ouyang P (2020). DN-GAN: Denoising generative adversarial networks for speckle noise reduction in optical coherence tomography images. Biomed Signal Process Control.

[14] Kafieh R, Rabbani H (2013) Optical coherence tomography noise reduction over learned dictionaries with introduction of complex wavelet for noise reduction, SPIE Proc. on Wavelets and Sparsity XV, San Diego, California, United States, 8858, 10.1117/12.2026520.

[15] Gopinath K, Rangrej SB, Sivaswamy J (2017) A deep learning framework for segmentation of retinal layers from OCT images, in 2017 4th IAPR Asian Conference on Pattern Recognition (ACPR), IEEE, pp. 888–893., 10.1109/ACPR.2017.121.

[16] Pekala M, Joshi N, Liu TA, Bressler NM, DeBuc DC, Burlina P (2019). Deep learning based retinal OCT segmentation. Comput Biol Med.

[17] Masood S, Fang R, Li P, Li H, Sheng B, Mathavan A, Wang X, Yang P (2019). Automatic choroid layer segmentation from optical coherence tomography images using deep learning. Sci Rep.

[18] Lee CS, Baughman DM, Lee AY (2017). Deep learning is effective for classifying normal versus age-related macular degeneration OCT images. Ophthalmology Retina.

[19] Alqudah AM (2020). AOCT-NET: a convolutional network automated classification of multiclass retinal diseases using spectral-domain optical coherence tomography images. Med Biol Eng Compu.

[20] Yoo TK, Choi JY, Kim HK (2021). Feasibility study to improve deep learning in OCT diagnosis of rare retinal diseases with few-shot classification. Med Biol Eng Compu.

[21] Miller A, Blott B (1992). Review of neural network applications in medical imaging and signal processing. Med Biol Eng Compu.

[22] Yoo TK, Choi JY, Seo JG, Ramasubramanian B, Selvaperumal S, Kim DW (2019). The possibility of the combination of OCT and fundus images for improving the diagnostic accuracy of deep learning for age-related macular degeneration: a preliminary experiment. Med Biol Eng Compu.

[23] Hsu S-H, Cao Y, Huang K, Feng M, Balter JM (2013). Investigation of a method for generating synthetic CT models from MRI scans of the head and neck for radiation therapy. Phys Med Biol.

[24] Kim J, Glide-Hurst C, Doemer A, Wen N, Movsas B, Chetty IJ (2015). Implementation of a novel algorithm for generating synthetic CT images from magnetic resonance imaging data sets for prostate cancer radiation therapy. International Journal of Radiation Oncology Biology Physics.

[25] Shin H-C, Tenenholtz NA, Rogers JK, Schwarz CG, Senjem ML, Gunter JL, Andriole KP, Michalski M (2018). Medical image synthesis for data augmentation and anonymization using generative adversarial networks. International workshop on simulation and synthesis in medical imaging.

[26] Xiao G, Brady M, Noble JA, Zhang Y (2002). Segmentation of ultrasound B-mode images with intensity inhomogeneity correction. IEEE Trans Med Imaging.

[27] Fiorini S, Ballerini L, Trucco E, Ruggeri A (2014) Automatic Generation of Synthetic Retinal Fundus Images. in MIUA, pp. 7–12, 10.2312/stag.20141238

[28] Costa P, Galdran A, Meyer MI, Abràmoff MD, Niemeijer M, Mendonça AM, Campilho A (2017) Towards adversarial retinal image synthesis, arXiv preprint arXiv:1701.08974/> 10.1109/TMI.2017.2759102 28981409

[29] Costa P, Galdran A, Meyer MI, Niemeijer M, Abràmoff M, Mendonça AM, Campilho A (2017). End-to-end adversarial retinal image synthesis. IEEE Trans Med Imaging.

[30] E. S. Varnousfaderani, W.-D. Vogl, J. Wu, B. S. Gerendas, C. Simader, G. Langs, S. M. Waldstein, and U. Schmidt-Erfurth, "Improve synthetic retinal OCT images with present of pathologies and textural information," in Medical Imaging 2016: Image Processing, 2016, vol. 9784: International Society for Optics and Photonics, p. 97843V. 10.1117/12.2217399.

[31] Serranho P, Maduro C, Santos T, Cunha-Vaz J, Bernardes R (2011) Synthetic oct data for image processing performance testing," in 2011 18th IEEE International Conference on Image Processing, IEEE, 401–404., 10.1109/ICIP.2011.6116534

[32] Montuoro A, Waldstein SM, Gerendas B, Langs G, Simader C, Schmidt-Erfurth U (2014). Statistical retinal OCT appearance models. Invest Ophthalmol Vis Sci.

[33] Kulkarni P, Lozano D, Zouridakis G, Twa M (2011) A statistical model of retinal optical coherence tomography image data, in 2011 Annual International Conference of the IEEE Engineering in Medicine and Biology Society, IEEE, pp. 6127–6130., 10.1109/IEMBS.2011.6091513 10.1109/IEMBS.2011.6091513 22255737

[34] Danesh H, Maghooli K, Dehghani A, Kafieh R (2020). Automatic production of synthetic labelled OCT images using an active shape model. IET Image Proc.

[35] O'Brien S, Ghita O, Whelan PF (2009) Segmenting the left ventricle in 3D using a coupled ASM and a learned non-rigid spatial model

[36] Zhu Y, Williams S, Zwiggelaar R (2007). A hybrid ASM approach for sparse volumetric data segmentation. Pattern Recognit Image Anal.

[37] Heimann T, Meinzer H-P (2009). Statistical shape models for 3D medical image segmentation: a review. Med Image Anal.

[38] Wang Z, Lim G, Ng WY, Keane PA, Campbell JP, Tan GSW, Schmetterer L, Wong TY (2021). Generative adversarial networks in ophthalmology: what are these and how can they be used?. Curr Opin Ophthalmol.

[39] Zha X, Shi F, Ma Y, Zhu W, Chen X (2019) Generation of retinal OCT images with diseases based on cGAN, in Medical Imaging 2019: Image Processing, 10949: International Society for Optics and Photonics, p. 1094924,doi: 10.1117/12.2510967

[40] Zheng C, Xie X, Zhou K, Chen B, Chen J, Ye H, Li W, Qiao T (2020). Assessment of generative adversarial networks model for synthetic optical coherence tomography images of retinal disorders. Translational Vision Science & Technology.

[41] Kugelman J, Alonso-Caneiro D, Read SA, Vincent SJ, Chen FK, Collins MJ (2021) Data augmentation for patch-based OCT chorio-retinal segmentation using generative adversarial networks. Neural Computing and Applications, pp. 1–16, 10.1007/s00521-021-05826-w

[42] Van Assen HC, Danilouchkine MG, Frangi AF, Ordás S, Westenberg JJ, Reiber JH, Lelieveldt BP (2006). SPASM: a 3D-ASM for segmentation of sparse and arbitrarily oriented cardiac MRI data. Med Image Anal.

[43] Kroon D-J (2011) Segmentation of the mandibular canal in cone-beam CT data. Citeseer, p.69, 201110.3990/1.9789036532808

[44] Davidson JA, Ciulla TA, McGill JB, Kles KA, Anderson PW (2007). How the diabetic eye loses vision. Endocrine.

[45] Ashtari F, Ataei A, Kafieh R, Khodabandeh Z, Barzegar M, Raei M, Dehghani A, Mansurian M (2020) Optical Coherence Tomography in Neuromyelitis Optica spectrum disorder and Multiple Sclerosis: A population-based study. Multiple Sclerosis and Related Disorders, 102625, 10.1016/j.msard.2020.102625 10.1016/j.msard.2020.102625 33227631

[46] Mahmudi T, Kafieh R, Rabbani H, Mehri A, Akhlaghi M-R (2021). Evaluation of asymmetry in right and left eyes of normal individuals using extracted features from optical coherence tomography and fundus images. Journal of Medical Signals and Sensors.

[47] Cootes T BE, Graham J (2000) An introduction to active shape models. Image processing and analysis, 223–48

[48] Behaine CAR, Scharcanski J (2014). Remote visual monitoring of analogue meter displays using deformable models. IET Sci Meas Technol.

[49] Montazerin M, Sajjadifar Z, Pour EK, Riazi-Esfahani H, Mahmoudi T, Rabbani H, Movahedian H, Dehghani A (2021). Livelayer: a semi-automatic software program for segmentation of layers and diabetic macular edema in optical coherence tomography images. Sci Rep.

[50] Kafieh R, Danesh H, Rabbani H, Abramoff M, Sonka M (2013) Vessel segmentation in images of optical coherence tomography using shadow information and thickening of Retinal Nerve Fiber Layer, in 2013 IEEE International Conference on Acoustics, Speech and Signal Processing, IEEE, 1075–1079, 10.1109/ICASSP.2013.6637815

[51] Chen E, Looman M, Laouri M, Gallagher M, Van Nuys K, Lakdawalla D, Fortuny J (2010). Burden of illness of diabetic macular edema: literature review. Curr Med Res Opin.

[52] Montuoro A, Waldstein SM, Gerendas BS, Schmidt-Erfurth U, Bogunović H (2017). Joint retinal layer and fluid segmentation in OCT scans of eyes with severe macular edema using unsupervised representation and auto-context. Biomed Opt Express.

[53] Lee CS, Tyring AJ, Deruyter NP, Wu Y, Rokem A, Lee AY (2017). Deep-learning based, automated segmentation of macular edema in optical coherence tomography. Biomed Opt Express.

[54] Wang Z, Zhang W, Sun Y, Yao M, Yan B (2020) Detection of Diabetic Macular Edema in Optical Coherence Tomography Image Using an Improved Level Set Algorithm. BioMed Research International, 2020, 10.1155/2020/6974215. 10.1155/2020/6974215 PMC7210525 32420362

[55] Chiu SJ, Allingham MJ, Mettu PS, Cousins SW, Izatt JA, Farsiu S (2015). Kernel regression based segmentation of optical coherence tomography images with diabetic macular edema. Biomed Opt Express.

[56] Schlegl T, Waldstein SM, Bogunovic H, Endstraßer F, Sadeghipour A, Philip A-M, Podkowinski D, Gerendas BS (2018). Fully automated detection and quantification of macular fluid in OCT using deep learning. Ophthalmology.

[57] Roy AG, Conjeti S, Karri SPK, Sheet D, Katouzian A, Wachinger C, Navab N (2017). ReLayNet: retinal layer and fluid segmentation of macular optical coherence tomography using fully convolutional networks. Biomed Opt Express.

[58] Chen Z, Li D, Shen H, Mo H, Zeng Z, Wei H (2020). Automated segmentation of fluid regions in optical coherence tomography B-scan images of age-related macular degeneration. Opt Laser Technol.

[59] Mujat M, Chan RC, Cense B, Park BH, Joo C, Akkin T, Chen TC, De Boer JF (2005). Retinal nerve fiber layer thickness map determined from optical coherence tomography images. Opt Express.

[60] Hall-Beyer M (2000). GLCM texture: a tutorial. National Council on Geographic Information and Analysis Remote Sensing Core Curriculum.

[61] Necasova T, Svoboda D (2018) Visual and quantitative comparison of real and simulated biomedical image data, in Proceedings of the European Conference on Computer Vision (ECCV) Workshops, 0–0, 10.1007/978-3-030-11024-6_28.

